# Which non-infection related risk factors are associated with impaired proximal femur fracture healing in patients under the age of 70 years?

**DOI:** 10.1186/s12891-023-06539-y

**Published:** 2023-05-20

**Authors:** Sascha Halvachizadeh, David Paul Martin, Roman Pfeifer, Gerrolt Nico Jukema, Boyko Gueorguiev, Hans-Christoph Pape, Till Berk

**Affiliations:** 1 Department of Trauma, University Hospital Zurich, Raemistrasse 100, 8091 Zurich, Switzerland; 2 Harald-Tscherne Laboratory for Orthopedic and Trauma Research, University of Zurich, Sternwartstrasse 14, 8091 Zurich, Switzerland; 3 Department of Orthopedics and Rehabilitation, University of Wisconsin, 1685 Highland Ave, Madison, WI 53705 USA; 4 AO Research Institute Davos, Clavadelerstrasse 8, 7270 Davos, Switzerland

**Keywords:** Traumatic proximal femur fractures, Polytrauma, Nonunion, Delayed union, Risk factors, Non-geriatric

## Abstract

**Background/purpose:**

Impaired healing is a feared complication with devastating outcomes for each patient. Most studies focus on geriatric fracture fixation and assess well known risk factors such as infections. However, risk factors, others than infections, and impaired healing of proximal femur fractures in non-geriatric adults are marginally assessed. Therefore, this study aimed to identify non-infection related risk factors for impaired fracture healing of proximal femur fractures in non-geriatric trauma patients.

**Methods:**

This study included non-geriatric patients (aged 69 years and younger) who were treated between 2013 and 2020 at one academic Level 1 trauma center due to a proximal femur fracture (PFF). Patients were stratified according to AO/OTA classification. Delayed union was defined as failed callus formation on 3 out of 4 cortices after 3 to 6 months. Nonunion was defined as lack of callus-formation after 6 months, material breakage, or requirement of revision surgery. Patient follow up was 12 months.

**Results:**

This study included 150 patients. Delayed union was observed in 32 (21.3%) patients and nonunion with subsequent revision surgery occurred in 14 (9.3%). With an increasing fracture classification (31 A1 up to 31 A3 type fractures), there was a significantly higher rate of delayed union. Additionally, open reduction and internal fixation (ORIF) (OR 6.17, (95% CI 1.54 to 24.70, *p* ≤ 0.01)) and diabetes mellitus type II (DM) (OR 5.74, (95% CI 1.39 to 23.72, *p* = 0.016)), were independent risk factors for delayed union. The rate of nonunion was independent of fracture morphology, patient’s characteristics or comorbidities.

**Conclusion:**

Increasing fracture complexity, ORIF and diabetes were found to be associated with delayed union of intertrochanteric femur fractures in non-geriatric patients. However, these factors were not associated with the development of nonunion.

## Introduction

Intertrochanteric femur fractures account for 42% of all proximal femur fractures [1]. The majority of younger trauma patients sustain a proximal femur fracture due to a high-energy trauma [2]. This trauma mechanism is usually associated with additional injuries and leads to more complex fracture morphology that includes an increased risk of osteonecrosis and impaired fracture healing [3, 4]. Young male adults and elderly patients are at increased risk for intertrochanteric femur fractures [5]. These populations represent two different entities of patients, with their own risk factors and comorbidities, and are therefore worth looking at separately. Intertrochanteric proximal femur fractures are known for high mortality rates, increased complications and are usually accompanied by comorbidities such as osteoporosis and diabetes mellitus (DM) [6, 7]. Impaired healing is a feared complication with devastating outcomes for each patient [8, 9]. Two major treatment options exist: extramedullary and intramedullary implants [10, 11]. A substantial increase in the use of intramedullary nails (from 3% up to 67%) during the past two decades has been observed in the United States and Europe [12]. Previous studies have shown that most treatment failures of fractures of the proximal femur occur in unstable fracture patterns, after mal-reduction of the posteromedial cortex or in patients with reverse oblique fractures [13–17]. Most studies focus on geriatric fracture fixation and assess well known risk factors, such as infections or low-grade infections and advanced age for delayed fracture healing [13, 18, 19]. However, risk factors, others than infections and advanced age, for impaired healing of proximal femur fractures in non-geriatric adults are marginally assessed. This study aims to identify non-infectious related risk factors for impaired fracture healing of proximal femur fractures in non-geriatric trauma patients.

## Methods

### Ethical consideration

This retrospective cohort study was approved by the Cantonal Ethics Committee, Canton of Zurich review board (Basec No.:2020–00703). A written consensus of data collection was obtained from all patients during hospitalization.

### Study population

#### Inclusion criteria

This study included non-geriatric patients (aged 69 years and younger) who were treated between 2013 and 2020 at one academic Level 1 trauma center due to a proximal femur fracture (PFF). The arbitrary cut-off of 70 years was chosen by our in-hospital protocol for geriatric co management. The geriatric co-management is the interdisciplinary collaboration of geriatricians and trauma surgeons. The interdisciplinary consensus has decided to set the cut-off age to 70 years [20]. Only patients that had a complete follow up of at least one year after surgery where included.

#### Exclusion criteria

Patients with genetic disorders affecting the musculoskeletal system, patients with oncologic diseases regarding the proximal femur, such a pathological fractures, fractures not classifiable as intertrochanteric fractures with the AO-classification and patients with postoperative infections were excluded from this study. Postoperative infections represent one of the major risk factors for impaired fracture healing. Numerous studies have investigated this association [21–23]. The goal of this study was to analyze other risk factor, that are not related with postoperative local infection, as defined as non-infection related. Therefore, these patients were excluded from the present study. Patients with additional ipsilateral lower limb fractures, open injuries, associated nerve and vascular injuries, and pelvic injuries were excluded. Patients requiring a staged procedure or primary plate fixation were also excluded from this study.

### Treatment protocol

All patients were treated surgically with a femoral nail, using the institutional standard implant a Gamma 3 nail (Stryker). The standard approach in our institute is a closed reduction under radiological control and internal fixation (CRIF) with the patient in a supine position on a traction table. If the reduction is inadequate in either the anterior–posterior or the lateral view, the treating surgeon decides for open reduction and internal fixation (ORIF). In both cases, the entry point for the intramedullary implant is the tip of the trochanter. In cases where sufficient reduction cannot be maintained, cerclage wires (cable cerclage, 1.6 mm in diameter) may be used at the discretion of the treating surgeon. Postoperative rehabilitation includes full weight bearing, daily physiotherapeutic training, and optimized medical treatment. The follow-up appointments were performed after 6 weeks, 12 weeks, 6 months and 12 months. Radiographic imaging to assess the state of fracture healing was obtained at each appointment, focusing on persistent fracture lines, insufficient bone bridging, progressive deformity and the presence/absence of broken implants.

### Outcomes and definitions

The primary outcome of this study was impaired fracture healing. Impaired fracture healing was subsequently specified as delayed union and nonunion. Per in-house protocol, the present study defined delayed union as callus formation on 3 out of 4 cortices after 3 to 6 months, in the absence of secondary fracture or material loosening. Nonunion was defined in cases of lack of callus-formation after 6 months, or the requirement of revision surgery. Equivalent definitions have been described in the literature [24]. The PFF was classified using the AO-Classification. Further, only fractures that where classifiable as intertrochanteric fractures with the AO-System for proximal femur fractures, 31 A1-3, were included [25]. Polytrauma was defined as an injury severity score (ISS) of 16 points or higher.

### Statistical analysis

Continuous variables are summarized as a mean with standard deviation (± SD). Categorical variables are displayed as count and percentages. Two groups of continuous variables were compared using the students t-test. For groups of binary variables, the chi-square test was used. ANOVA was used when comparing more than two groups. A p-value below 0.05 was considered statistically significant. Regression analysis was performed with the primary outcome being impaired fracture healing. Multivariate regression analysis included variables of clinical significance, or risk factors that are associated with increased complication rates. All analyses were performed using R (R Core Team (2019). R: A language and environment for statistical computing. R Foundation for Statistical Computing, Vienna, Austria.)

## Results

This study included 150 non-geriatric trauma patients at a mean age of 54.15 ± 12.52 years. 45 (30.0%) patients were female. The leading comorbidities were arrhythmia that required oral anticoagulation drugs (OAK) (*N* = 38, 25.3%, most of these patients aged 50 years and above *N* = 33, 87.1%), followed by osteoporosis (*N* = 22, 14.7%, most of these patients aged 50 years and above *N* = 21, 95.5%). The overall length of stay (LOS) was 11.27 ± 9.16 days (Table 1). The most common fracture type, was AO 31A1 (*N* = 61, 40.7%), followed by AO type 31A2 (*N* = 48, 33.0%). Delayed union was observed in 32 (21.3%) patients and nonunion with subsequent revision surgery occurred in 14 (9.3%) (Table 2).

**Table 1:** Patients demographics and fracture classification

N	150
Sex = female, N (%)	45 (30.0)
Age (years), (mean (SD))	54.15 (12.52)
DM Type II, N (%)	13 (8.7)
Polytrauma (ISS > 16), N (%)	38 (25.3)
Osteoporosis, N (%)	22 (14.7)
LOS, days, (mean (SD))	11.27 (9.16)
ORIF, N (%)	49 ( 32.7)
Cerclage, N (%)	33 ( 22.0)

*N* number, *SD* Standard Deviation, *DM* diabetes mellitus, *LOS* Length of stay, *ISS* injury severity score, *ORIF* open reduction and internal fixation

**Table 2:** Fracture classification and impaired fracture healing

N	150
ORIF, N (%)	49 ( 32.7)
Cerclage, N (%)	33 ( 22.0)
Delayed union, N (%)	32 (21.3)
Nonunion, N (%)	14 (9.3)
AO type 31A1, N (%)	**61 ( 40.7)**
1.1	7 (4.7)
1.2	34 (22.7)
1.3	20 (13.3)
AO type 31A2, N (%)	**48 ( 32.0)**
2.1	14 ( 9.3)
2.2	16 ( 10.7)
2.3	18 ( 12.0)
AO type 31A3, N (%)	**41 ( 27.3)**
3.1	19 (12.7)
3.2	8 (5.3)
3.3	14 (9.3)

*N* number, *ORIF* open reduction and internal fixation, *AO* Arbeitsgemeinschaft für Osteosynthese fragen

ORIF and Cerclage usage, were significantly more often used in AO 31 A3 type fractures (*N* = 27, 65.9%) and (*N* = 16, 39.0%) when compared to AO 31 A1 (*N* = 4, 6.6%) / (*N* = 2, 3.3%) and AO 31 A2 (*N* = 18, 37.5%) / (*N* = 15, 31.2%). With an increasing fracture classification (31 A1 up to 31 A3 type fractures), there was a significantly higher rate of delayed union: AO 31 A1 (*N* = 6, 9.8%), AO 31 A2 (*N* = 12, 25.0%) and AO 31 A3 (*N* = 14, 34.1%), (*p* ≤ 0.01, Table 3). Patients with delayed union had a significantly higher rate of ORIF (*n* = 18, 56.2%) when compared with patients without delayed union (*n* = 31, 26.3%, *p* = 0.003). Further, patients with diabetes had higher rates of delayed union (*n* = 7, 21.9%) when compared with non-diabetic patients. Both AO type 31A2 and 31A3 fractures were associated with an increased risk for the development of delayed union (95% CI 1.05 to 8.87, *p* = 0.04; 95% CI 1.64 to 13.73, *p* = 0.004) when compared with AO type 31A1 fractures. Additionally, ORIF (OR 6.17, (95% CI 1.54 to 24.70, *p* ≤ 0.01)) and DM (OR 5.74, (95% CI 1.39 to 23.72, *p* = 0.016)), were independent risk factors for delayed union (Table 4). The rate of nonunion was independent from fracture morphology, patient characteristics or comorbidities (Table 5).

**Table 3:** Distribution according to the AO-classification

Groups according to the AO-classification	31A1	31A2	31A3	*p*-value
N	61	48	41	
Sex = female, N (%)	21 (34.4)	10 (20.8)	14 (34.1)	0.243
Age (years), (mean (SD))	53.52 (12.15)	53.85 (13.66)	55.44 (11.88)	0.738
ORIF, N (%)	4 (6.6)	18 (37.5)	27 (65.9)	** < 0.001**
Cerclage, N (%)	2 (3.3)	15 (31.2)	16 (39.0)	** < 0.001**
Delayed union, N (%)	6 (9.8)	12 (25.0)	14 (34.1)	**0.01**
Nonunion, N (%)	5 (8.2)	4 (8.3)	5 (12.2)	0.761
DM Type II, N (%)	3 (4.9)	3 (6.2)	7 (17.1)	0.078
Polytrauma (ISS > 16), N (%)	12 (19.7)	13 (27.1)	13 (31.7)	0.369
Osteoporosis, N (%)	6 (9.8)	10 (20.8)	6 (14.6)	0.273
Oncology, N (%)	2 (3.3)	2 (4.2)	6 (14.6)	0.055
LOS, days, (mean (SD))	10.77 (11.04)	10.19 (6.30)	13.29 (8.78)	0.242

*N* number, *SD* Standard Deviation, *DM* diabetes mellitus, *LOS* Length of stay, *ISS* injury severity score, *AO* Arbeitsgemeinschaft für Osteosynthese fragen, *ORIF* open reduction and internal fixation

**Table 4:** Delayed union univariate and multivariate regression analysis

	Odds Ratio	95% confidence Intervall low	95% confidence Intervall hight	*p*-value
**Univariate regression analysis:**				
Comparison AO Classification 31A:				
2.1–3	3.056	1.052	8.874	**0.04**
3.1–3	4.753	1.644	13.739	**0.004**
**Multivariate regression analysis:**				
Comparison AO Classification 31A:				
2.1–3	1.686	0.505	5.636	0.396
3.1–3	1.404	0.353	5.584	0.63
Sex = female	0.419	0.134	1.303	0.133
Age (years)	1.028	0.983	1.075	0.231
ORIF	6.176	1.544	24.703	**0.01**
Cerclage	0.414	0.101	1.694	0.22
DM Type II	5.749	1.393	23.729	**0.016**
Polytrauma (ISS > 16)	2.092	0.671	6.52	0.203
Osteoporosis	3.111	0.968	10.003	0.057
Oncology	2.315	0.447	12	0.317

*AO* Arbeitsgemeinschaft für Osteosynthesefragen, *ORIF* open reduction and internal fixation, *DM* diabetes mellitus, *ISS* injury severity score

**Table 5:** Nonunion univariate and multivariate regression analysis

	Odds Ratio	95% confidence Intervall low	95% confidence Intervall hight	*p*-value
**Univariate regression analysis:**				
Comparison AO Classification 31A:	Ref			
2.1–3	1.018	0.258	4.018	0.979
3.1–3	1.556	0.42	5.756	0.508
**Multivariate regression analysis:**				
Comparison AO Classification 31A:	Ref			
2.1–3	0.713	0.156	3.263	0.662
3.1–3	0.657	0.116	3.712	0.634
Sex = female	0.528	0.117	2.376	0.405
Age (years)	1.01	0.955	1.068	0.733
ORIF	2.72	0.469	15.76	0.264
Cerclage	0.475	0.068	3.296	0.451
DM Type II	4.428	0.847	23.148	0.078
Polytrauma (ISS > 16)	3.032	0.755	12.175	0.118
Osteoporosis	1.97	0.419	9.266	0.391
Oncology	1.471	0.132	16.447	0.754

*AO* Arbeitsgemeinschaft für Osteosynthesefragen, *ORIF* open reduction and internal fixation, *DM* diabetes mellitus, *ISS* injury severity score

## Discussion

Intertrochanteric proximal femur fractures are associated with increased mortality, increased complications and often comorbidities such as osteoporosis and DM. This study aimed to assess non-infectious related risk factors for delayed union and nonunion of PFF in non-geriatric trauma patients and found the following points:

1. AO 31 A3 type fractures have an increased rate of impaired healing
2. Additional injuries are not associated with delayed union or nonunion
3. ORIF and DM are independent risk factors for delayed union, but not for nonunion.

Certain risk factors for impaired bone healing include local infections, advanced age, multiple comorbidities, smoking, non-steroidal anti-inflammatory usage, sex, metabolic disease and nutritional deficiencies [26–28]. Further, fracture pattern and location, degree of displacement, severity of soft tissue injury, quality of surgical treatment, degree of bone loss and presence/absence of infection have also been reported as risk factors for impaired bone healing [29]. Intertrochanteric fractures are known to have an excellent blood supply and good cancellous bone in the intertrochanteric region; therefore, nonunion is uncommon [30]. Generally, intertrochanteric fractures treated with internal fixation heal [9, 18]. It is well known, that failure of fracture fixation and nonunion can occur due to poor bone quality, unfavorable fracture patterns, suboptimal internal fixation and delayed treatment of patients [13, 14, 31–33]. The most common fracture types for nonunion of the proximal femur are 31 A 1.1, 2.3 and 3.3 [33, 34]. These findings were comparable with the findings in this study. The reported incidence of intertrochanteric fractures resulting in nonunion ranges between 1–2% [35]. The investigated proximal fractures caused by high energy trauma in non-geriatric populations are known to be more difficult to reduce and more prone to complications [36, 37]. These finding could be an explanation for the deviation from our results.

The present results show that delayed union rate but not non-union was associated with fracture morphology. In younger trauma patients, it may therefore be appropriate to wait longer before initiating revision surgeries, even in cases of clinical or radiological suspicion of delayed fracture healing. As definitions of delayed union and nonunion are vague, there is no agreement on the exact timing when the diagnosis should be made [38]. Various studies come to different timeframes regarding the diagnosis of nonunion, with a variety between 6–8 weeks, 3 months and up to 6 months [39–41]. Since the clinical evaluation of fracture healing is a combination of both radiographic and clinical findings [42], patients with unremarkable clinical and radiological findings at 6 – 12 weeks postoperatively, and similar results for the rest of the consultations, were considered cured/healed.

This study showed that additional injuries seemed not to be associated with impaired bone healing. In femoral shaft fracture, ISS has been reported to not be associated with nonunion [43]. Controversially, other studies found nonunion in long bone fractures as a common problem in multiply injured patients [44–48]. Early final stabilization of long bone fractures during the first 24 h after the initial trauma has been shown to be the best treatment option for major fractures [49–51]. In this study, ORIF and DM serve as independent risk factors for impaired bone healing in non-geriatric trauma patients. Comparable findings are known for subtrochanteric femur fractures with risk factors including chronic diseases such as diabetes mellitus [52]. Nonunion is reported to occur in 95% of cases with unstable fractures with loss of medial calcar continuity following ORIF [31]. Our data showed that nonunion had no association to the reduction technique used and was therefore independent of ORIF or CRIF.

A systematic review further concluded that the reduction technique is not associated with nonunion in femoral neck fractures [53]. Conversely, it was been well studied that minimally invasive surgeries of the femur had shorter healing times and lower re-operation rates [54–56]. Our results may indicate that the 31A3 fracture types require special surgical attention for a beneficial outcome. The focus should be on an optimal surgical reduction, which can be achieved by an ORIF and the support of cerclage wires, if necessary. The femoral subtrochanteric region has a critical blood supply and is mainly composed of cortical bone [57]. Lack of medial cortical support, varus malreduction and residual displacement after reduction have been described as potential risk factors for nonunion of subtrochanteric femur fractures [58–60].

### Strength & limitations

The retrospective design provides certain well-known limitations. One might argue that this study has an increased risk for type 2 error. We believe, that based on the standardized treatment protocol and the comparability of the study groups, the presented results provide some evidence for the identification of risk factors for nonunion. There is a lack of standardized quantification methods for the assessment of the quality of fracture reduction in the clinical setting. Therefore, documentation of the quality of fracture reduction is missing. This important factor is incorporated in our in-hospital protocol by utilizing the best-possible reduction prior nail fixation on the surgeons’ best judgement.

Occult infection is a potential cause of implant failure of a proximal femur fracture and should be considered [61]. In this cohort, there were no infections diagnosed in patients with nonunion or who required revision surgery, since patients with postoperative infections where excluded from this study. In addition, very limited information on tobacco consumption was available at the time of data analysis. The known negative effect of smoking on fracture healing could therefore not be taken into account in this study.

## Conclusion

In this study, increasing fracture complexity, ORIF, and diabetes were found to be associated with delayed union of intertrochanteric femur fractures in non-geriatric patients. These variables, however, did not show a significant association with the development of non-union.

## Data Availability

The collected data will be stored securely in our institute for 10 years. During this period, they are still available on request. After the 10 years, the data will be deleted. However, all the datasets analyzed or generated during this study will be available from corresponding author on reasonable request.

## References

[1] Adeyemi A, Delhougne G. Incidence and Economic Burden of Intertrochanteric Fracture: A Medicare Claims Database Analysis. JB JS Open Access. 2019;4(1):e0045. 10.2106/JBJS.OA.18.00045. 10.2106/JBJS.OA.18.00045 PMC6510469 31161153

[2] Rogmark C, Kristensen MT, Viberg B, Ronnquist SS, Overgaard S, Palm H (2018). Hip fractures in the non-elderly-Who, why and whither?. Injury-Int J Care Injured.

[3] Slobogean GP, Sprague SA, Scott T, Bhandari M (2015). Complications following young femoral neck fractures. Injury-Int J Care Injured.

[4] Stockton DJ, Lefaivre KA, Deakin DE, Osterhoff G, Yamada A, Broekhuyse HM (2015). Incidence, Magnitude, and Predictors of Shortening in Young Femoral Neck Fractures. J Orthop Trauma.

[5] Robinson CM, Courtbrown CM, McQueen MM, Christie J. Hip fractures in adults younger than 50 years of age. Epidemiology and results. Clin Orthopaed Related Res. 1995(312):238–46. 7634609

[6] Melton LJ, Gabriel SE, Crowson CS, Tosteson ANA, Johnell O, Kanis JA (2003). Cost-equivalence of different osteoporotic fractures. Osteoporos Int.

[7] Norris R, Parker M (2011). Diabetes mellitus and hip fracture: A study of 5966 cases. Injury-Int J Care Injured.

[8] Kyle RF, Cabanela ME, Russell TA, Swiontkowski MF, Winquist RA, Zuckerman JD, et al., editors. Fractures of the proximal part of the femur. 1994 Instructional Course Lectures, at the 61st Annual Meeting of the American-Academy-of-Orthopaedic-Surgeons; 1995 1994; New Orleans, La 1995. 7797861

[9] Kyle RF, Gustilo RB, Premer RF (1979). Analysis of six hundred and twenty-two intertrochanteric hip fractures. J Bone Joint Surg-Am Vol.

[10] Domingo L, Cecilia D, Herrera A, Resines C (2001). Trochanteric fractures treated with a proximal femoral nail. Int Orthop.

[11] Leung K, So W, Shen W, Hui P. Gamma nails and dynamic hip screws for peritrochanteric fractures. A randomised prospective study in elderly patients. J Bone Joint Surg Brit Vol. 1992;74(3):345–51. 10.1302/0301-620X.74B3.1587874 1587874

[12] Anglen JO, Weinstein JN, American Board of Orthopaedic Surgery Research C. Nail or plate fixation of intertrochanteric hip fractures: changing pattern of practice: a review of the American Board of Orthopaedic Surgery Database. JBJS. 2008;90(4):700–7. 10.2106/JBJS.G.00517 18381305

[13] Haidukewych GT, Israel TA, Berry DJ (2001). Reverse obliquity fractures of the intertrochanteric region of the femur. J Bone Joint Surg-Am Vol.

[14] Sarathy MP, Madhavan P, Ravichandran KM. Nonunion of intertrochanteric fractures of the femur. Treatment by modified medial displacement and valgus osteotomy. J Bone Joint Surg-Brit Vol. 1995;77B(1):90–2. 7822404

[15] Wu CC, Shih CH, Chen WJ, Tai CL (1998). Treatment of cutout of a lag screw of a dynamic hip screw in an intertrochanteric fracture. Arch Orthop Traum Su.

[16] Bartonicek J, Skala-Rosenbaum J, Dousa P (2003). Valgus intertrochanteric osteotomy for malunion and nonunion of trochanteric fractures. J Orthop Trauma.

[17] Haidukewych GJ, Berry DJ (2003). Salvage of failed internal fixation of intertrochanteric hip fractures. Clin Orthop Relat Res.

[18] Baumgaertner MR, Solberg BD (1997). Awareness of tip-apex distance reduces failure of fixation of trochanteric fractures of the hip. J Bone Joint Surg-Brit Vol.

[19] Parker MJ (1994). Prediction of fracture union after internal fixation of intracapsular femoral neck fractures. Injury.

[20] Halvachizadeh S, Gröbli L, Berk T, Jensen KO, Hierholzer C, Bischoff-Ferrari HA (2021). The effect of geriatric comanagement (GC) in geriatric trauma patients treated in a level 1 trauma setting: A comparison of data before and after the implementation of a certified geriatric trauma center. PLoS ONE.

[21] Struijs PA, Poolman RW, Bhandari M (2007). Infected nonunion of the long bones. J Orthop Trauma.

[22] Yin P, Ji Q, Li T, Li J, Li Z, Liu J (2015). A Systematic Review and Meta-Analysis of Ilizarov Methods in the Treatment of Infected Nonunion of Tibia and Femur. PLoS ONE.

[23] Walter N, Rupp M, Krückel J, Alt V (2022). Individual and commercially available antimicrobial coatings for intramedullary nails for the treatment of infected long bone non-unions - a systematic review. Injury.

[24] Willems A, van der Jagt OP, Meuffels DE (2019). Extracorporeal shock wave treatment for delayed union and nonunion fractures: a systematic review. J Orthop Trauma.

[25] Müller ME, Nazarian S, Koch P, Schatzker J. The comprehensive classification of fractures of long bones: Springer Science & Business Media; 2012.

[26] Pountos I, Georgouli T, Pneumaticos S, Giannoudis PV (2013). Fracture non-union: Can biomarkers predict outcome?. Injury-Int J Care Injured.

[27] Copuroglu C, Calori GM, Giannoudis PV (2013). Fracture non-union: Who is at risk?. Injury-Int J Care Injured.

[28] Rajasekaran S, Giannoudis PV (2012). Open injuries of the lower extremity: Issues and unknown frontiers. Injury-Int J Care Injured.

[29] Bishop JA, Palanca AA, Bellino MJ, Lowenberg DW (2012). Assessment of Compromised Fracture Healing. J Am Acad Orthop Sur.

[30] Angelini M, McKee MD, Waddell JP, Haidukewych G, Schemitsch EH (2009). Salvage of Failed Hip Fracture Fixation. J Orthop Trauma.

[31] Mariani EM, Rand JA. Nonunion of intertrochanteric fractures of the femur following open reduction and internal fixation. Results of second attempts to gain union. Clin Orthopaed Related Res. 1987(218):81–9. 3568500

[32] Boyd HB, Lipinski SW (1957). Nonunion of trochanteric and subtrochanteric fractures. Surg Gynecol Obstetr.

[33] Dhammi IK, Jain AK, Singh AP, RehanUl H, Mishra P, Jain S (2011). Primary nonunion of intertrochanteric fractures of femur: An analysis of results of valgization and bone grafting. Indian J Orthopaed.

[34] Spiegel P. Fracture and dislocation compendium. Orthopaedic trauma association committee for coding and classification. J Orthop Trauma. 1996;10:1–154. 8814583

[35] Altner P. Reasons for failure in treatment of intertrochanteric fractures. 1982.

[36] Rogmark C, Kristensen MT, Viberg B, Rönnquist SS, Overgaard S, Palm H (2018). Hip fractures in the non-elderly—who, why and whither?. Injury.

[37] Amini MH, Feldman JJ, Weinlein JC (2017). High complication rate in young patients with high-energy intertrochanteric femoral fractures. Orthopedics.

[38] Bhandari M, Guyatt GH, Swiontkowski MF, Tornetta Iii P, Sprague S, Schemitsch EH (2002). A lack of consensus in the assessment of fracture healing among orthopaedic surgeons. J Orthop Trauma.

[39] Nayak NK, Schickendantz MS, Regan WD, Hawkins RJ (1995). Operative treatment of nonunion of surgical neck fractures of the humerus. Clin Orthop Relat Res.

[40] Cadet ER, Yin B, Schulz B, Ahmad CS, Rosenwasser MP (2013). Proximal humerus and humeral shaft nonunions. J Am Acad Orthopaed Surgeons.

[41] McQueen MM (2008). Nonunions of the proximal humerus: their prevalence and functional outcome. J Trauma Acute Care.

[42] Hak DJ, Fitzpatrick D, Bishop JA, Marsh JL, Tilp S, Schnettler R (2014). Delayed union and nonunions: Epidemiology, clinical issues, and financial aspects. Injury-Int J Care Injured.

[43] Taitsman LA, Lynch JR, Agel J, Barei DP, Nork SE (2009). Risk factors for femoral nonunion after femoral shaft fracture. J Trauma Acute Care.

[44] Lu C, Miclau T, Hu D, Marcucio RS (2007). Ischemia leads to delayed union during fracture healing: a mouse model. J Orthop Res.

[45] Garcia P, Histing T, Holstein J, Klein M, Laschke M, Matthys R (2013). Rodent animal models of delayed bone healing and non-union formation: a comprehensive review. Eur Cell Mater.

[46] Balogh ZJ, Reumann MK, Gruen RL, Mayer-Kuckuk P, Schuetz MA, Harris IA (2012). Advances and future directions for management of trauma patients with musculoskeletal injuries. Lancet.

[47] Dahabreh Z, Dimitriou R, Giannoudis PV (2007). Health economics: a cost analysis of treatment of persistent fracture non-unions using bone morphogenetic protein-7. Injury.

[48] Hannon M, Hadjizacharia P, Chan L, Plurad D, Demetriades D (2009). Prognostic significance of lower extremity long bone fractures after automobile versus pedestrian injuries. J Trauma Acute Care.

[49] Grundnes O, Reikeraas O (2000). Effects of macrophage activation on bone healing. J Orthop Sci.

[50] Nowotarski PJ, Turen CH, Brumback RJ, Scarboro JM (2000). Conversion of external fixation to intramedullary nailing for fractures of the shaft of the femur in multiply injured patients. JBJS.

[51] Kazakos KJ, Verettas DJ, Tilkeridis K, Galanis VG, Xarchas KC, Dimitrakopoulou A (2006). External fixation of femoral fractures in multiply injured intensive care unit patients. Acta Orthop Belg.

[52] Napoli N, Schwartz AV, Palermo L, Jin JJ, Wustrack R, Cauley JA (2013). Risk factors for subtrochanteric and diaphyseal fractures: the study of osteoporotic fractures. J Clin Endocrinol Metab.

[53] Ghayoumi P, Kandemir U, Morshed S (2015). Evidence based update: open versus closed reduction. Injury.

[54] Zlowodzki M, Williamson S, Cole PA, Zardiackas LD, Kregor PJ (2004). Biomechanical evaluation of the less invasive stabilization system, angled blade plate, and retrograde intramedullary nail for the internal fixation of distal femur fractures. J Orthop Trauma.

[55] Schandelmaier P, Partenheimer A, Koenemann B, Grün OA, Krettek C (2001). Distal femoral fractures and LISS stabilization. Injury.

[56] Markmiller M, Konrad G, Südkamp N. Femur–LISS and distal femoral nail for fixation of distal femoral fractures: are there differences in outcome and complications? Clin Orthopaed Related Res. 2004;426:252–7. 10.1097/01.blo.0000141935.86481.ba 15346082

[57] Krappinger D, Wolf B, Dammerer D, Thaler M, Schwendinger P, Lindtner RA (2019). Risk factors for nonunion after intramedullary nailing of subtrochanteric femoral fractures. Arch Orthop Traum Su.

[58] Beingessner DM, Scolaro JA, Orec RJ, Nork SE, Barei DP (2013). Open reduction and intramedullary stabilisation of subtrochanteric femur fractures: A retrospective study of 56 cases. Injury-Int J Care Injured.

[59] Park SH, Kong GM, Ha BH, Park JH, Kim KH (2016). Nonunion of subtrochanteric fractures: Comminution or Malreduction. Pakistan J Med Sci.

[60] Shukla S, Johnston P, Ahmad MA, Wynn-Jones H, Patel AD, Walton NP (2007). Outcome of traumatic subtrochanteric femoral fractures fixed using cephalo-medullary nails. Injury-Int J Care Injured.

[61] Haidukewych G, Berry DJ (2005). Salvage of failed treatment of hip fractures. J Am Acad Orthop Sur.

